# Gender Difference in sidE eFfects of ImmuNotherapy: a possible clue to optimize cancEr tReatment (G-DEFINER): study protocol of an observational prospective multicenter study

**DOI:** 10.2340/1651-226X.2024.24179

**Published:** 2024-04-21

**Authors:** Rosalba Miceli, Hanna Eriksson, Giuseppe Lo Russo, Salvatore Alfieri, Maria Moksnes Bjaanæs, Filippo Pietrantonio, Loris De Cecco, Arsela Prelaj, Claudia Proto, Johan Franzén, Deirdre McDonnell, José Javier Berenguer Pina, Teresa Beninato, Laura Mazzeo, Patrizia Giannatempo, Elena Verzoni, John Crown, Åslaug Helland, Alexander J Eustace

**Affiliations:** a Unit of Biostatistics for Clinical Research, Department of Epidemiology and Data Science, Fondazione IRCCS Istituto Nazionale dei Tumori, Milan, Italy; b Department of Oncology-Pathology, Karolinska Institutet, Stockholm, Sweden; c Theme Cancer, unit of Head-Neck-, Lung-, and Skin Cancer Karolinska University Hospital-Solna, Stockholm, Sweden; d Thoracic Oncology Unit, Medical Oncology Department, Fondazione IRCCS Istituto Nazionale dei Tumori, Milan, Italy; e Head and Neck Unit, Medical Oncology Department, Fondazione IRCCS Istituto Nazionale dei Tumori, Milan, Italy; f Department of Oncology, Oslo University Hospital, Oslo, Norway; g Medical Oncology Department, Fondazione IRCCS Istituto Nazionale dei Tumori, Milan, Italy; h Integrated biology of rare tumors, Department of Experimental Oncology, Fondazione IRCCS Istituto Nazionale dei Tumori, Milan, Italy; i Department of Medical Oncology, St Vincent’s University Hospital, Dublin, Ireland; j School of Biotechnology, Life Sciences Institute, Dublin City University, Dublin,Ireland; k Radium Hospital/Oncology, University of Oslo, Institute of Clinical medicine, Oslo, Norway

**Keywords:** Cancer, immune checkpoint inhibitors, immune-related adverse events, sex- and gender differences, prospective trial

## Abstract

**Background:**

Immune checkpoint inhibitors (ICIs) have significantly improved outcomes in various cancers. ICI treatment is associated with the incidence of immune-related adverse events (irAEs) which can affect any organ. Data on irAEs occurrence in relation to sex- differentiation and their association with gender-specific factors are limited.

**Aims:**

The primary objective of the G-DEFINER study is to compare the irAEs incidence in female and male patients who undergo ICI treatment. Secondary objectives are: to compare the irAEs incidence in pre- and postmenopausal female patients; to compare the irAEs incidence in female and male patients according to different clinical and gender-related factors (lifestyle, psychosocial, and behavioral factors). Exploratory objectives of the study are to compare and contrast hormonal, gene-expression, SNPs, cytokines, and gut microbiota profiles in relation to irAEs incidence in female and male patients.

**Methods and Results:**

The patients are recruited from Fondazione IRCCS Istituto Nazionale dei Tumori, Italy, St Vincent’s University Hospital, Ireland, Oslo University Hospital, Norway, and Karolinska Insitutet/Karolinska University Hospital, Sweden. The inclusion of patients was delayed due to the Covid pandemic, leading to a total of 250 patients recruited versus a planned number of 400 patients. Clinical and translational data will be analyzed.

**Interpretation:**

The expected outcomes are to improve the management of cancer patients treated with ICIs, leading to more personalized clinical approaches that consider potential toxicity profiles. The real world nature of the trial makes it highly applicable for timely irAEs diagnosis.

## Introduction

Immune checkpoint inhibitors (ICIs) have significantly improved outcomes in various cancers [1]. At present, primary ICI targets include cytotoxic T-lymphocyte-associated protein 4 (CTLA-4), programmed cell death protein-1 (PD-1) and its ligand programmed cell death-ligand 1 (PD-L1) [2]. Recent approval for Lymphocyte-activation gene 3 (LAG-3) targeting ICI therapy in melanoma has increased the range of approved ICI treatments [3]. Many studies have reported that ICI treatment results in favorable overall survival (OS) and progression-free survival (PFS) benefits and in some cases is associated with long-term durable responses [4, 5].

However, the increasing use of ICI treatment is associated with unique immune-related adverse events (irAEs). Different factors regulate tumor immunity and response to ICI treatment, including internal factors such as the tumor and its microenvironment, genetic and epigenetic factors, host immunity, and the role of the gut microbiota. Further external gender-related host factors should also be considered, such as factors including lifestyle (diet, smoking, physical activity), psycho-social (socio-economic, emotional), and behavioral (living arrangement, emotional wellbeing) [6].

Previous evidence has demonstrated that these gender-related factors may have a direct correlation with stress [7–9]. Stress has been shown to have a direct impact on a cancer patient’s inflammatory response [7, 10]. These lifestyle, psycho-social, and behavioral factors which constitute our lifetime experiences may result in stress associated changes to our inflammatory responses and may impact response to ICI therapy [11] and on the development of irAEs.

Although any organ system can be affected, irAEs most commonly involve the gastrointestinal tract, endocrine glands, skin, and liver [12, 13]. IrAEs can occur anytime during a course of ICI treatment but usually occur within the first few weeks up to 6 months after treatment initiation. However, in some cases irAEs can occur even after completion of treatment. Remarkably, irAEs have been associated with a positive response to treatment, indicating their potential as predictive biomarkers [14, 15].

Evidence suggests that sex-based differences in immune responses contribute to disease outcome and response to ICI [16–18] treatment. Sex, defined by differential organization of chromosomes, reproductive organs, and sex steroid levels, is a biological variable profoundly affecting immune response, and influencing irAEs types, frequency, and severity. Women tend to have stronger immune responses and react more vigorously to infections and vaccines and are more likely to develop systemic autoimmune diseases [19]. As an example, during the COVID-19 pandemic, although the SARS-COV-2 infection rates were similar between sexes, men experienced more severe illness, resulting in a higher mortality rate than that observed in women [20, 21].

Genes on the X chromosome play a significant role in immune response regulation [22]. It has been suggested that there is an association between the increasing number of X chromosomes an individual has, and a higher risk of developing autoimmunity-related diseases [23]. Since genes influence the risk of developing autoimmune diseases even in the absence of ICI treatment (including myasthenia and thyroid and polyglandular disease [24]); large genome-wide association studies (GWAS) are needed to establish a relationship between genetic factors and irAEs risk in ICI treatment.

The production of cytokines and chemokines by innate immune cells also differs between sexes. For example, neutrophils from men produce a greater amount of tumor-necrosis factor (TNF) than women [9]; hormones also affect immune cells, where they impact anti-tumor immunity and treatment response [19]. For example, estrogen may have both stimulatory or inhibitory effects, while testosterone suppresses immune responses. Regions of X chromosomes that are active in synergy with sex hormones can make a difference in modeling the type, extent, and duration of inflammatory responses [19].

Therefore the interplay of genetics, biology, and hormonal influences might contribute to irAE inequalities between female (F) and male (M) patients. Further, psycho-social factors, socioeconomic context, and lifestyle choices could also play a role, but studies on their association with irAEs in ICI treatment are limited. However, an extensive study of gender-based inequalities in health [27] reported that some measures related to structural (e.g. age, family arrangement, occupation), lifestyle (smoking, drinking, physical activity, weight), and psycho-social context (mainly related to stress) are differently distributed between F and M, and most of them are related to health inequalities.

Despite thorough data collection on irAEs in registrational studies, there is limited reporting on sex differentiation and its association with gender-specific factors.

The study’s main hypothesis is that F and M patients who receive ICI treatment will have different risks of irAE events. What affects the risk of irAE in an individual patient especially in regard to sex is insufficiently understood, making the objective of this study important. As pointed out by Les et al., [28] long-term prospective cohorts and real-life studies are needed to assess potential irAE biomarkers. Our study will comprehensively analyze sex as a biomarker of irAE incidence in ICI treatment. Exploring irAEs incidence in relation to patients’ sex is of clinical relevance, and developing tools to identify irAE high-risk patients would be of help to optimize patient selection for ICI treatments with the aim of minimizing toxicity.

## Methods

### Objectives

The primary objective was to compare the irAEs incidence in female and male patients who undergo ICI treatment.

Secondary objectives were to compare the irAEs incidence in pre- and postmenopausal female patients; to estimate the irAEs incidence according to different clinical features, and gender-related factors.

Exploratory objectives of the study were to compare and contrast hormonal, gene-expression, SNPs, cytokines, and gut microbiota profiles in relation to irAEs incidence in female and male patients.

### Study population

The G-DEFINER study population consists of patients who underwent ICI treatment in oncology clinics from four European hospitals: Fondazione IRCCS Istituto Nazionale dei Tumori (INT) (Italy), St Vincent’s University Hospital (Ireland), Oslo University Hospital – (Norway), and Karolinska Institutet, Karolinska University Hospital (Sweden).

### Eligibility criteria

The G-DEFINER study consisted of patients who were ≥18 years old and had a Eastern Cooperative Oncology Group (ECOG) Performance Status of 0–2. Patients must have had adequate bone marrow, liver, and renal function and a life expectancy of at least 12 weeks.

Patients must have histologically confirmed melanoma, lung, head and neck, urogenital, and other solid tumors, for example, MSI-high characterized by the presence of microsatellite instability. We enrolled cancer patients with any disease stage from the above-listed cancers and any treatment setting including neoadjuvant, adjuvant, advanced disease, maintenance, and compassionate use. Eligible ICI-containing regimens, included single-agent ICI, dual ICI combinations, ICI and chemotherapy, and ICI and radiotherapy combinations. Exclusion criteria included those patients who were unable to understand the clinical trial information. Further exclusions comprised those patients who were not eligible for ICI-containing regimens as per established clinical practice, or those cancer patients who were either pregnant or actively breastfeeding.

### Study design

This is a multicenter prospective observational study.

**Figure 1:**
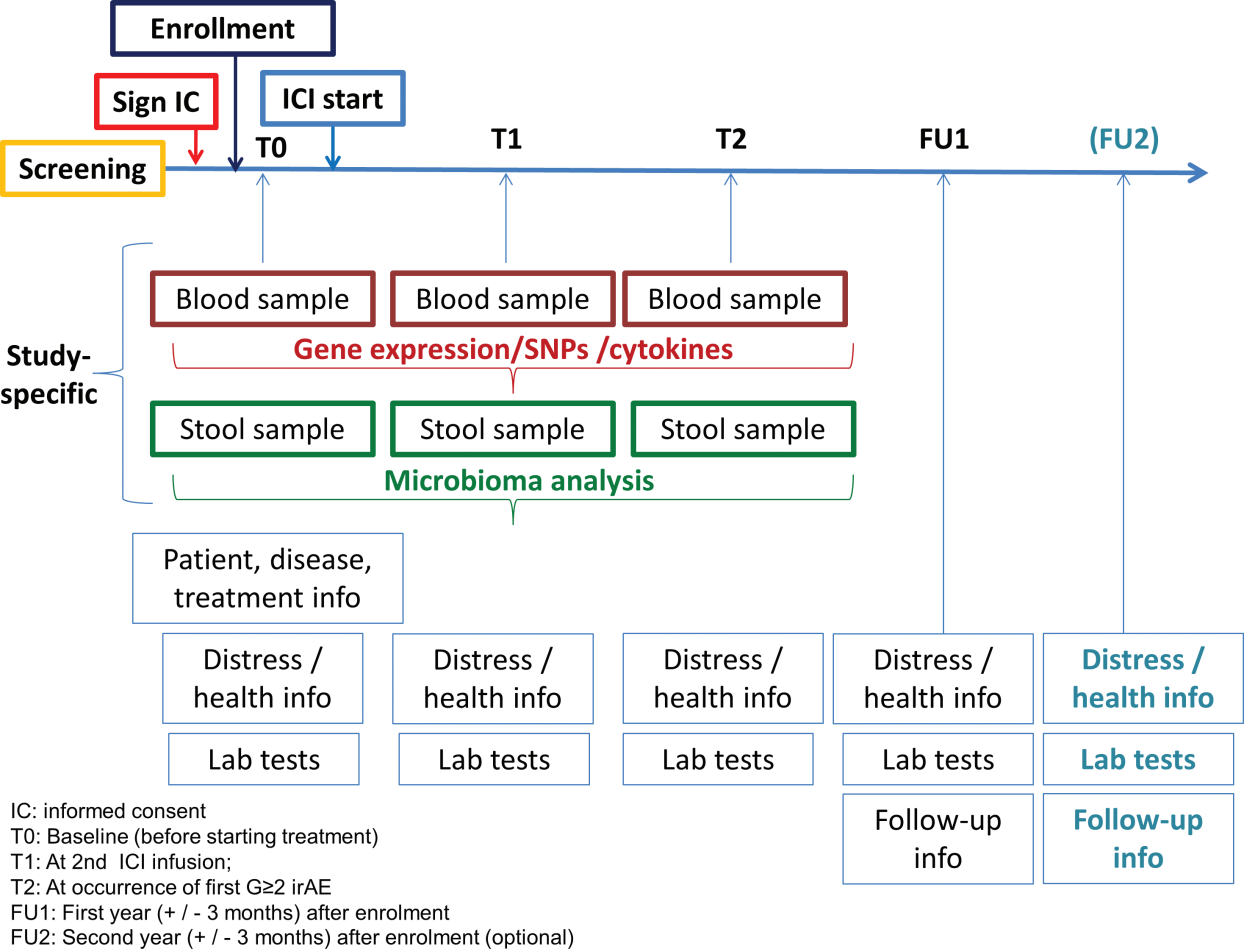
Overall study design in the G-DEFINER trial.

Recruited patients are treated with ICI containing regimens according to usual clinical practice (see Figure 1 for overall study design). The original scheduled plan was to recruit a total number of 400 patients in four centers, with 24 months recruitment plus 12 months follow-up. However, the COVID-19 pandemic negatively impacted on patients’ accrual, resulting in a total accrual of 250 patients.

### Treatment and clinical procedures

Treatment regimens are administered as per clinical prescription.

Patients underwent physical examinations, laboratory tests, and disease assessments using CT or other imaging methods following standard clinical practices at each Center. IrAEs are recorded and graded using the Common Terminology Criteria for Adverse Events (CTCAE) v. 5.0. Patients experiencing irAEs may continue treatment as per clinical indications.

### Data and Bio-sample collection

**Figure 2:**
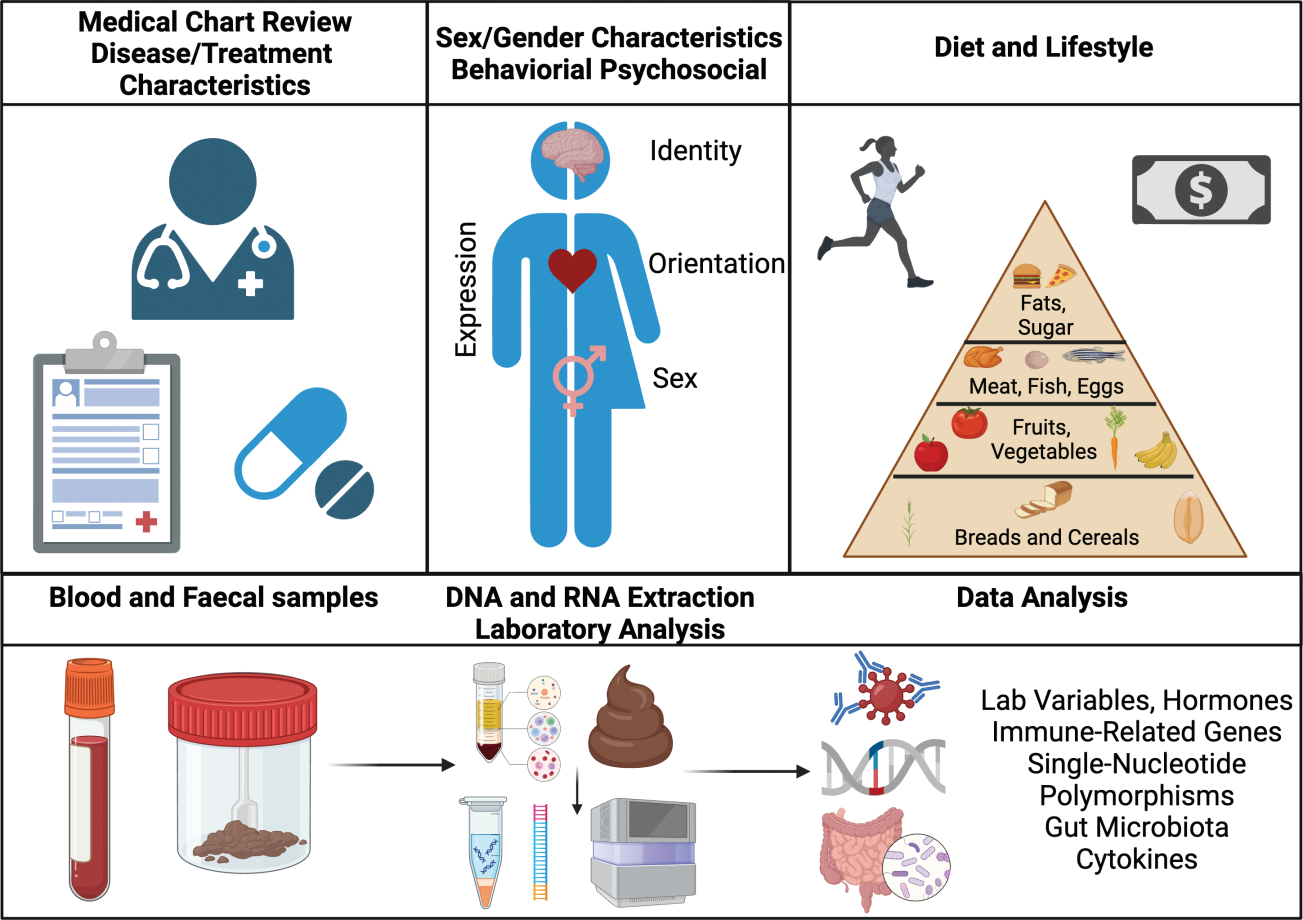
Data assessed in the G-DEFINER study. Figure created using BioRender.com.

Data collection was standardized across Centers as per G-DEFINER specific SOPs to prevent bias. Data were entered by all sites onto tailored Case Report Form (CRF) and electronic CRFs. The study collects data from Oncology Units treating eligible patients in the participating centers, primarily during routine checks. Gender related data, based on the results shown in Ref. [27], behavioral (e.g. living arrangement, occupation, socio-economic), lifestyle (e.g. smoking, drinking, physical activity, diet), and psycho-social factors (e.g. social support, emotional, and physical issues, stress). General health levels are assessed, including mobility, self care, usual activities, pain anxiety (Figure 2). Full details on CRFs can be retrieved on https://zenodo.org/record/4142787; in particular, gender-related variables are reported in ‘CRF – 02 Registration and baseline’ (Sections 7 and 8), ‘CRF 05 – Questionnaires distress and health’ and ‘CRF – 10 – Diet form’.

Biosamples were collected to derive data for exploratory studies aimed at comparing and contrasting hormonal, gene-expression, SNPs, cytokines, and gut microbiota profiles in relation to irAEs incidence in female and male patients. Blood samples for gene-expression profiling and cytokine analysis, as well as samples for gut microbiota assessment, are taken at baseline (before ICI starts) and at second ICI infusion. SNPs blood samples are taken at baseline only. Additional samples could be drawn at occurrence of the first irAE of Grade ≥ 2. Blood samples are collected during routine medical examinations using standard drawing procedure. Stool samples are obtained from patients at home, with the provision of dedicated kits.

### Endpoints and statistical analyses

Collected data will be statistically described according to their qualitative and quantitative nature and results displayed using tables, listings, and figures. IrAEs will be tabulated, including their overall number and the number of patients reporting irAEs, grouped by CTCAE class and grading, and according to sex, ICI treatment, and tumor type.

The *primary end-point* was to compare the incidence of grade ≥ 2 irAEs in female and male patients who undergo ICI treatment. As the G-DEFINER study will inevitably recruit most patients from the metastatic setting, which will contribute to the increased incidence of mortality (competing event) which may occur prior to the incidence of any irAEs, we will use survival analysis methods in a competing risks setting. The comparison F versus M will be performed by estimating the grade ≥ 2 crude cumulative incidence of the event, and the grade ≥ 2 irAEs sub-distribution hazard ratio (HR) using a univariable Fine and Gray model [29]. We will perform this analysis by taking into account the discrepancies between the two sex groups. For this purpose, we will apply the ‘matching weight’ method [30] according to which the patients’ weights will be estimated as a function of a model-based propensity score, taking into account all the discrepancy factors (including Country, for instance). This method is effective, as the model can include only the sex variable, and the between patients discrepancies are taken into account by the weighting system; thus the model will easily accomplish the ‘ten events per variable’ rule, preserving the statistical power of the analysis.

The *secondary end-points* were: (a) to compare grade ≥ 2 irAEs incidence in pre- and postmenopausal female patients. In this comparison of pre- and postmenopausal female patients the ‘matching weight’ method [30] will be applied to adjust for confounding factors. (b) To compare grade ≥ 2 irAEs incidence in female and male patients according to different clinical and gender-related factors. The analysis of these end-points will be performed using survival analysis methods in a competing risks setting, where death in the absence of irAEs will be regarded as a competing event. A further hypothesis could be that female or male patients could experiment different burdens of irAEs in relation to the gender-related variables, that is, there could be an interaction between sex and these variables. The study power is not sufficient to detect as statistically significant all the interactions, hence these analyses are to be interpreted as exploratory and the results should be interpreted with caution.

*Exploratory end-points:* to compare and contrast routine lab tests, gene-expression, SNPs, cytokines, and gut microbiota profiles in relation to irAEs incidence in female and male patients. These analyses will be performed to identify the features associated with irAEs occurrence according to sex. Sex-specific irAEs signatures will be developed by performing class comparison (univariable) and class prediction (multivariable) analyses. In class prediction analysis, since the number of features to be investigated is high, after class comparison pre-filtering, variable selection techniques will be used. Feature selection will be performed using effective methods, such as the ‘LASSO’ (least absolute shrinkage and selection operator) [31, 32] with stability selection [33], widely and effectively used with high dimensional data such as those produced in genome research. This algorithm performs both variable selection by penalizing less important features and regularization in order to enhance the prediction accuracy. Stability selection provides finite sample control for some error rates of false discoveries. This method can be also applied with survival data, as in our case, in which Grade ≥ 2 irAEs (main endpoint) are to be analyzed taking into account their time of occurrence, death as competing event, and censored times. The selected features will be then included in Grade ≥ 2 irAEs statistical models, where the ‘matching weight’ method [30] will be applied to adjust for confounding factors.

*Sample size calculation:* This calculation refers to the primary end-point analysis, that is, the estimation of grade ≥ 2 irAEs sub-distribution HR to compare female and male patients using a univariable Fine and Gray model, taking into account the discrepancies between the two groups by means of the ‘matching weight’ method [30]. A sample size of 250 patients will achieve a 80% power at a 5% significance level to detect values of the HR between 1.60 and 1.67, under the hypothesis of unequal ratios F/M, as shown in the Table 2.

**Table 1:** Explanation of biological sample assessments conducted on serial blood and fecal samples.

Gene-expression analysis, SNPs analysis, cytokines	Blood samples are taken to identify: - immune-related components or biological pathways - germline SNPs - cytokines with the hypothesis that these factors are associated with the development of irAEs in relation to sex.
Gut microbiota analysis	Stool samples are collected to analyze gut microbiota, with the goal of performing DNA sequencing to identify components associated with the development of irAEs in relation to sex.
Routine lab tests	Blood samples are taken in order to identify blood biomarkers associated with development of irAEs in relation to sex.

**Table 2:** Hazard ratio (HR) that can be estimated under different scenarios defined by expected proportion of grade ≥ 2 irAEs and female to male recruitment.

Overall proportion of grade ≥ 2 irAEs*	F/M ratio	HR
50%	45%/55%	1.65
60%	45%/55%	1.58
50%	40%/60%	1.67
60%	40%/60%	1.59

F: female; M: male; HR: hazard ratio.

* Values estimated in preliminary analyses.

## Discussion

This is the first prospective study aimed at defining the sex role in regard to the frequency of irAE incidence in patients who receive ICI therapy. The results from this study may aid the development of tools for individual irAEs prediction based on sex, gender, clinical, genetic, immunological, and microbiom features. G-DEFINER seeks to address sex disparities in the field of oncology, by investigating whether such differences contribute to irAEs occurrence.

The expected outcomes are to improve the management of patients who will undergo treatment with ICI therapy, leading to more personalized clinical approaches that consider potential toxicity profiles. The ‘real world’ (outside experimental clinical trial setting) nature of the study makes it highly applicable for timely irAEs diagnosis. Furthermore, the study aims to promote ‘sex based’ approaches in cancer treatment, particularly those involving ICIs, with the potential to positively impact clinical management and reduce healthcare system costs.

## Data Availability

The data generated in this study are not publicly available due to information that could compromise patient privacy or consent but are available upon reasonable request from the corresponding author.
